# Field evaluation of WALS truck-mounted A1 super duty mist sprayer® with VectoBac® WDG against *Aedes aegypti* (Diptera:Culicidae) populations in Manatee County, Florida

**DOI:** 10.1007/s42452-021-04893-x

**Published:** 2022-01-06

**Authors:** Katie F. Williams, Samantha Ramirez, Christopher R. Lesser

**Affiliations:** Manatee County Mosquito Control District, 2317 2nd Ave W, Palmetto, FL 34221 USA

**Keywords:** Integrated vector management, Ovitrap, Egg surveillance, Adult surveillance, Insecticides, Resistance, Organic, *Bacillus thurigiensis israelensis*

## Abstract

*Aedes aegypti*, the Dengue and Zika vector, is a domestic mosquito that is difficult to control. The challenge lies in the mosquito’s preference to lay its eggs in cryptic habitats such as fence post openings, buckets and bird baths, cups. Additionally, current methods of control are labor-intensive. We employed the WALS strategy to evaluate the operational efficacy of applying VectoBac WDG using a truck-mounted mister to control local populations of *Ae. aegypti* in urban settings with difficult-to-reach larval mosquito habitats. Our study was conducted in Cortez (Manatee County), Florida, USA (27° 27’ N, 82° 40’ W). We selected two study sites, one untreated control (21.9 ha) and one treatment site (23.1 ha) where historical data have shown high populations of *Ae. aegypti* based on weekly ovitrap surveillance. Weekly BGs and ovitraps (10–15 traps/site) were deployed to monitor adult population dynamics. A total of 50 bioassay jars were placed in the field each afternoon of the *Bti* application (8 application events) at fixed locations to collect droplets and gauge efficacy. We found significant reduction in female adults (*P* = *0.0002)* and landing rate counts (*P* = *0.0058)* as a result of treatment. Larval bioassays during the eight applications confirmed *Bti* deposit in a variety of coverage types regardless of placement in the yards. WALS applications with a truck-mounted mister can be effective at reducing disease carrying mosquito populations in residential areas and can be implemented in an integrated vector management program.

**Supplementary Information:**

The online version contains supplementary material available at 10.1007/s42452-021-04893-x.

## Background

The incidence of infectious diseases is increasing as globalization and urbanization become more prominent driving the distribution of vectors and introduction of diseases to non-endemic areas [1–3]. In recent years, exotic arthropod-borne viruses such as Dengue (DENV), Zika (ZIKV) and Chikungunya (CHIKV) have had sporadic outbreaks throughout Florida causing largely febrile illness, some leading to severe complications [2, 4]. The lack of vaccines and effective antiviral treatments against these arboviruses and their diseases, emphasizes the need for integrated mosquito control to reduce transmission and disease outbreaks [1, 5]. The primary vectors, *Aedes (Stegomyia) aegypti* (Linnaeus, 1762) and *Aedes (Stegomyia) albopictus* (Skuse, 1894) are challenging to control due to their exploitation of diverse and hidden receptacles or foliage as egg-laying sites [2]. Preventive measures of these diseases are heavily reliant on effective vector control actions and elimination of larval habitats.

The expanding distribution of *Ae. aegypti* and *Ae. albopictus* throughout tropical, sub- tropical, and temperate regions places an increasing number of the world’s population at risk of contracting vector-borne viruses [2, 6]. The *Aedes* expansion into non-endemic areas has been noted throughout history and has greatly impacted Florida over the last 40 years [7–9]. Additionally, the risk of introduction of arboviruses by infected travelers returning from tropical countries which are endemic to pathogens such as DENV has increased the chances of imported and locally acquired cases [4, 8].

One of the most burdensome mosquito-borne diseases is dengue infecting 390 million people and incurring global costs of $8.9 billion in 2013 alone [6, 10]. In a decade where other infectious disease mortality rates have decreased, dengue rates notably increased by 48% causing alarm for other viruses transmitted by *Ae. aegypti* or *Ae. albopictus* [7]. Moreover, DENV transmission does not appear to be slowing down as all regions in the world were affected in 2019 [11]. In the last five years, 64% (371/581) of travel associated DENV cases in Florida were acquired in 2019 [12] and while travel cases significantly decreased in 2020 due to COVID travel restrictions [13], locally acquired cases are on an upward trend in the USA [12]. During 2020, locally acquired DENV cases in the contiguous USA increased from 20 to 80 cases, a 4 × fold increase, with most cases (86%) coming from South Florida [12, 14] likewise, Puerto Rico had a 31.5 × fold increase from 28 to 756 locally acquired DENV cases compared to the prior year, resulting in 76% (756/1002) of its locally transmitted cases in the last five years [12]. Approximately 77% (70/91) of locally acquired DENV cases in the last five years in Florida were acquired in 2020 with cases increasing from 18 to 70, a 3.9 × fold increase over the last year [12]. The prevalence of these mosquito-borne diseases is not declining, and their ability to spread in Florida’s conducive climate is increasingly clear, indicating the need for stronger interventions at controlling these *Aedes* populations.

Residential landscapes are constantly growing and changing bringing in additional natural and artificial breeding sites for *Ae. aegypti* and *Ae. albopictus t*o exploit. Targeting these urbanized mosquitoes is challenging as the many potential egg-laying sites in a single plot of land makes search and destroy or direct hand application by inspector's time consuming and laborious and provides potential impediments to typical control methods [15, 16]. In addition, continuous applications of a small list of available products have compromised efficacy due to an increased prevalence of insecticide resistance [17, 18].

The prevalence and spread of arbovirus outbreaks throughout the United States and Florida requires IVM programs to target various life cycles in order to reduce adult mosquito populations [9, 19, 20, 21]. One key component of IVM is diligent surveillance of current insecticide resistant levels in mosquito populations ensuring effective control [22]. In Florida, genetic and phenotypic studies have shown that local populations *of Ae. aegypti* throughout the state have decreasing susceptibility levels to the pyrethroid class of insecticides [17, 22, 23]. Genetic analyses have also showed that within Manatee County alone, there is a range of permethrin resistance levels among adult *Ae. aegypti* populations [23]. With a limited arsenal of insecticides to choose from, pyrethroids and organophosphates (OPs) being the two classes of chemicals available to treat adult mosquitoes for vector control, product rotation can be somewhat challenging [17, 22, 23]. In addition to genetic and resistance testing, MCMCD monitors mortality in field caged mosquito truck ultra-low volume trials (ULV) which gives key insight into the field efficacy one would see with operational ULV missions as described in Williams et. al. [23].

With an increasing necessity to explore strategies targeting immature stages of a mosquito’s life cycle, popular target-specific biological larvicides like *Bacillus thuringiensis israelensis* (*Bti)* are commonly used as these products are a bio-rational alternative approved by the World Health Organization (WHO) and the Organic Materials Review Institute (OMRI). The minimal non-target effects and organic origin of this bio-larvicide provides an advantage when used alongside traditional insecticides in an IVM approach [20, 24, 25, 26].

Utilizing Valent BioSciences’ WALS approach, area-wide larviciding of VectoBac WDG, involves applying an emulsified larvicide with a low-volume sprayer so the droplets are capable of drifting into difficult-to-find and/or access to larval mosquito habitats. Application methods in the WALS approach include backpack sprayers for targeted coverage, aerial application for large spray blocks and vehicle-mounted sprayers for wide-area coverage of specific blocks [27, 28].

The present study describes a large-scale field trial evaluating the operational efficacy of WALs strategy, applying VectoBac WDG with truck-mounted equipment (A1 Super Duty Mister) under an open-field setting as well as operational trials in urban and suburban locations to control local populations of *Ae. aegypti.* The objective of this study was to evaluate the operational efficacy of applying VectoBac WDG to reduce *Ae. aegypti* populations in one urban environment of Manatee County.

## Methods

### Ethics statement

All pesticide applications were made by county mosquito control agencies under the authority of Florida Statutes Chapter 388 and the Florida Administrative Code Chapter 5E-13. These studies did not involve endangered or protected species. No specific permits were required for the described field studies.

### Study sites

This study was carried out by MCMCD in Cortez, Florida (27° 27’ N, 82° 40’ W) during May through September 2020. The trial consisted of biweekly field work in two test sites and a separate site for the droplet characterization of the bio-larvicide. The first study site, Cortez Village contained the plot that was used for the eight application replicates. Cortez Village (27° 28’ N, 82° 41’ W) was 23.1 ha consisting of nine residential streets, four residential avenues and 148 parcels (houses with surrounding yard). Most of the parcels in the study site contained neglected vegetation with cryptic habitats such as planters, buckets, boats and rain gutters that tend to be abundant egg-laying habitat for *Ae. aegypti.* The second study site was located approximately 3,158 m east of the treatment site and was used for the control site (27° 29’ N, 82° 39’ W). The control site was 21.9 ha consisting of four residential streets, one residential avenue and 96 parcels. Residential properties in this site were similar to that of the treatment location, with some homes containing buckets, toys and neglected yards with an accumulation of various containers. The site used for droplet characterization was a 14.1 ha open field at Manatee Fruit Co in Sunny Shores, Cortez (27° 28’ N, 82° 40’ W). The selection of the above-mentioned sites followed the district’s historical *Ae. aegypti* surveillance data collected from 2010 to the present from these locations as sites demonstrating high mosquito populations. Average weather conditions throughout the trial period (May-Sept 2020) were 31.96 °C, 4.12 mm of rainfall and wind speeds of 13.16 kph (Weather Underground, wunderground.com, Brookhaven, GA).

### Pesticide

VectoBac WDG bacterial larvicide is a water-dispersible granular formulation of *Bacillus thuringiensis* subsp. *israelensis* (strain AM65-52) containing 3,000 International Toxin Units (ITU) per milligram of product. This product was chosen because it consists of only *Bti* and food-grade (U.S. EPA list 4) inert ingredients as well as the environmental profile, high target specificity, fast results; mortality typically occurring within 2–24 h and no record of insecticide resistance to this active ingredient by target mosquito populations [19, 26, 28, 29, 30]. VectoBac WDG is registered for direct application in cryptic water holding containers, the preferred oviposition sites of *Ae. aegypti*, as well as for wide-area low-volume treatments of larval habitats through various spray application techniques [15].

### Equipment

We chose the A1 Super Duty Larvicide Sprayer (Adapco, Sanford, FL) with the Micronair AU5000 atomizer (Micron Sprayers Limited, Herefordshire, UK). The Micronair atomizer uses a rotating wire gauze cylinder to produce spray droplets in the extremely fine to fine (EF/VF/F) size classification ensuring the ideal droplet spectrum for the bio-larvicide. The Micronair is driven by airflow from three 69.85 mm fan blades set at 55 degrees [30] that can be adjusted to produce the correct droplet size for a particular application and a 20-mesh screen. The A1 mister has a 379 L polyethylene tank and a 20 hp twin cylinder electric start Honda GX 630 engine (Honda Engines Group, Alpharetta, Ga).

### Droplet characterization

**Fig. 1:**
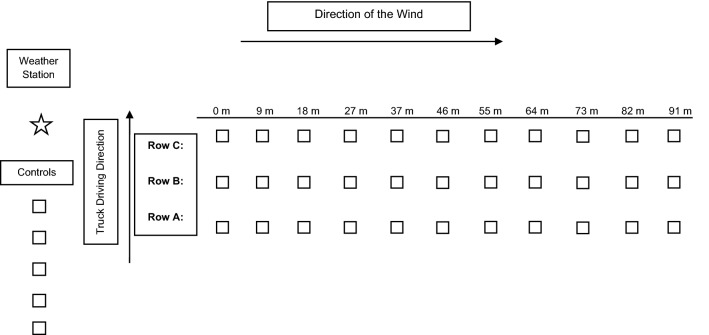
Map of the droplet characterization grid, including weather station, the locations of the control sampling stations (n=5), the location of the rows (n=3), bioassay jars at different distances from the spray path (0-91m), Sunny Shores, Cortez, Florida, 2020

Droplet spectrum was characterized using the BacDrop™ (Valent BioSciences LLC, Libertyville, IL) which provides droplet sizes and densities. Droplet collection stations were established starting from zero to 91 m downwind of the spray path in three rows 30 m apart (A, B and C [Fig. 1]). At each station one CD jewel case with a 51 × 76 mm Kromekote C2C white card (Unisource, Québec) attached with a box clip was set along with an identifying flag. The CD cases were used to prevent moisture from soaking into the Kromekote cards. The glossy surface of the card was place facing up. Each station also included a 177 ml polystyrene bioassay jar (United States Plastic Corp®, Lima, OH). Additionally, five control stations were set up upwind of the spray path (Fig. 1). Wind direction and speed were monitored in five second intervals using a Kestrel® 4500 NV Model Pocket Weather® Tracker (KestrelMeters.com, Boothwyn, PA).

To identify droplet spectrum and produce contrasting pictures on the cards, 4.54 kg of VectoBac WDG was mixed with 272 g of red food dye (Sensient Colors LLC, St. Louis, MO) and 37.85 L of tap water. The flow rate was set to 11.4 L/min with an application speed of 16 km/h, a swath of 91.4 m and a targeted application rate of 560 g/ha (representing mid-label rates on the USA pesticide label). The truck drove upwind and as close to the stations as possible, starting approximately 121 m before row A began and 121 m after row C ended to ensure proper dispersion throughout the entire treatment plot. Ten minutes post- application, the Kromekote cards were placed in plastic bags for safe transfer and later analysis. The bioassay jars were collected and brought back to the laboratory to assess the presence of the biolarvicide droplets inside each container. The jars were filled with 100 ml of reverse-osmosis water and mixed to suspend *Bti* residue. Approximately 20 L3 lab-reared *Ae. aegypti* larvae were then added to each jar (treatment and control) and monitored up to 48 h to monitor larvae mortality.

### Mosquito rearing

Lab-reared, susceptible *Ae. aegypti* eggs were obtained from the United States Department of Agriculture (USDA, Gainesville) strain in 2016 and a population has been maintained in an insectary at the district’s facilities. Adult mosquitoes were held in 30 × 30 × 30 cm collapsible cages (BioQuip Products, Rancho Dominguez, CA) with constant access to a 10% sucrose solution. To maintain the colony, the female mosquitoes were given cotton balls soaked with defibrinated bovine blood (HemoStat Laboratories, Dixon, CA) with a parafilm membrane and heated at 38–45° C (standard temperature used at MCMCD) for 45 min once a week. Three days after blood-feeding, egg bowls containing three labeled seed germination papers were placed in the cages to collect eggs. The egg bowls remained for three days and were subsequently dried out for no less than 24 h prior to hatching.

Insectary conditions were maintained under a controlled temperature (26 °C ± 1), a relative humidity (RH) of 75% ± 5% and a photoperiod of 14:10 (L:D) h [5]. Larvae were reared in 58.4-cm-long × 41.3-cm-wide × 15.2-cm-high plastic containers containing 3 L of reverse-osmosis filtered water and fed 30 ml of a 3:2 liver powder and brewer’s yeast slurry (500 ml reverse-osmosis filtered water, 15 g liver powder, 10 g of brewer’s yeast; MP Biomedical, Solon, OH) until the larvae were third instar then removed to be used in the larval jar bioassays.

### Operational trials

**Fig. 2:**
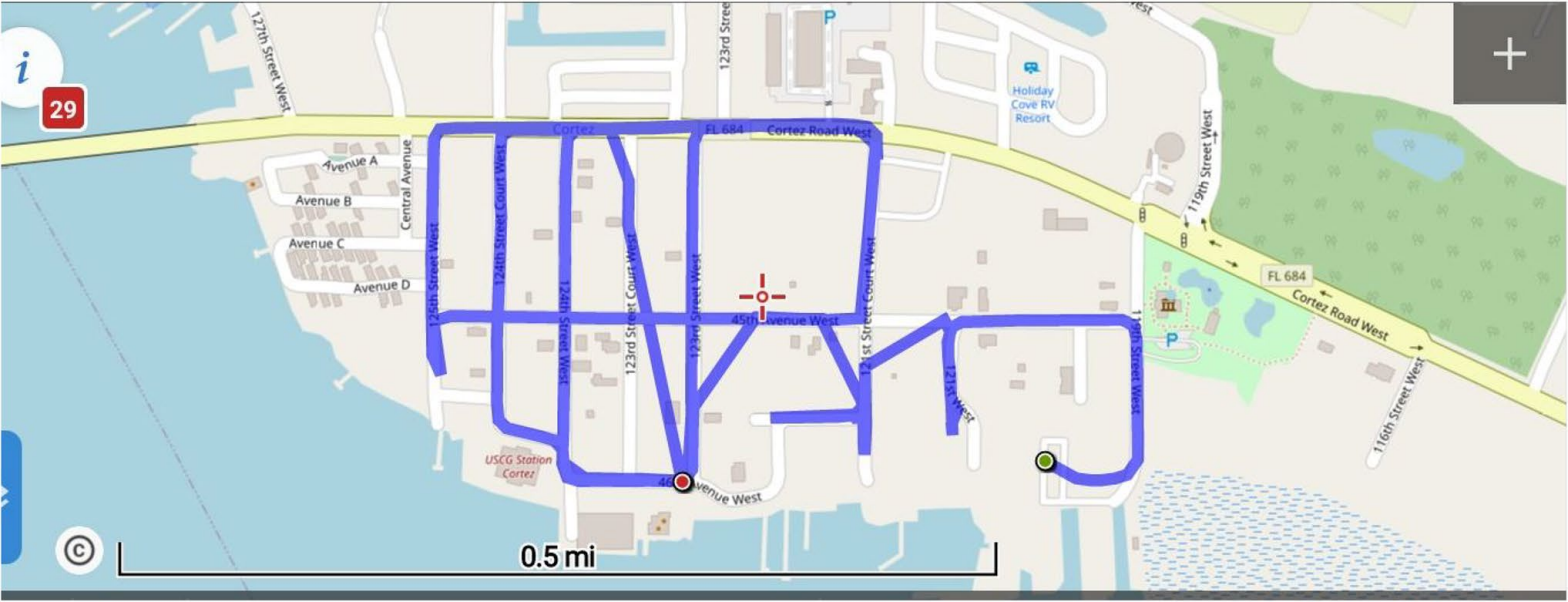
Map of the treatment site and the eight WALS *Bti *applications using the A1 Super Duty Mister in Cortez Village, FL

A total of eight WALS *Bti* applications were conducted using the A1 Mister Super Duty Mister in the treatment site. The experiment was set up as previously described with a flow rate of 11.4 L/min. VectoBac WDG was mixed under agitation with tap water at a rate of 95 L to 11 kg of product. The vehicle was driven at an average speed of 16 km/h for a final application rate of 560 g/ha (Fig. 2). Meteorological data were monitored using the Manatee County Booster Station Tower-KFLCORTE4 for temperature, wind direction, wind speed and relative humidity at the time of application 21:00 h. The operational trials were performed June through August 2020 (Week of Year 23–38). Four applications were conducted one day/week, after the four weekly treatments, the remaining four treatments were every other week (following manufacturer recommendations). All pesticide applications were made by MCMCD licensed spray applicators.

**Fig. 3:**
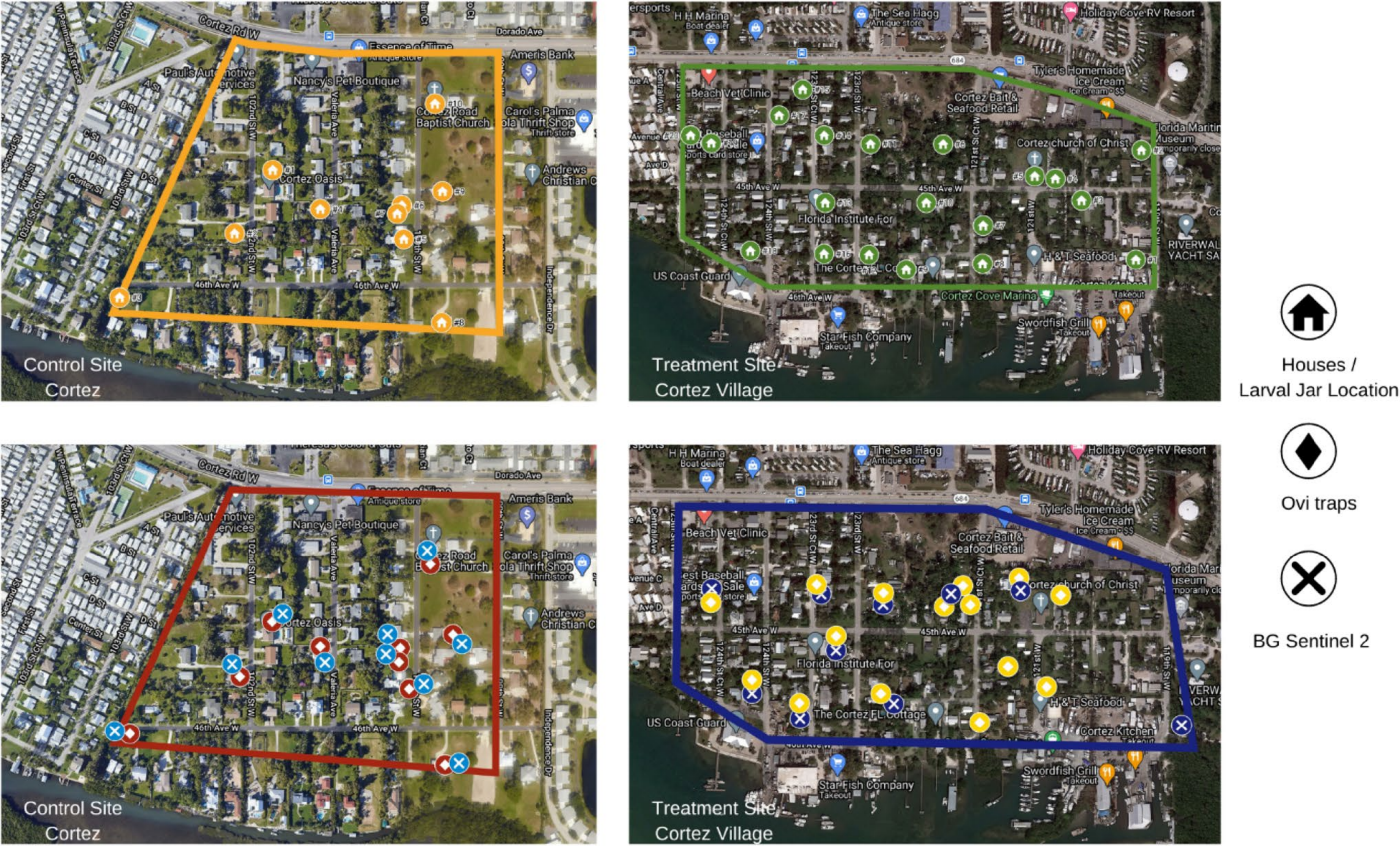
Aerial overview of experimental sites in Cortez, Florida, Manatee County. Top Left: Control site larval jar locations. Top Right: Treatment site larval jar locations. Bottom Left: Control site surveillance locations with ovitraps and BG-Sentinel 2 Traps. Bottom Right: Treatment site surveillance locations with ovitraps and BG-Sentinel 2 Traps

**Fig. 4:**
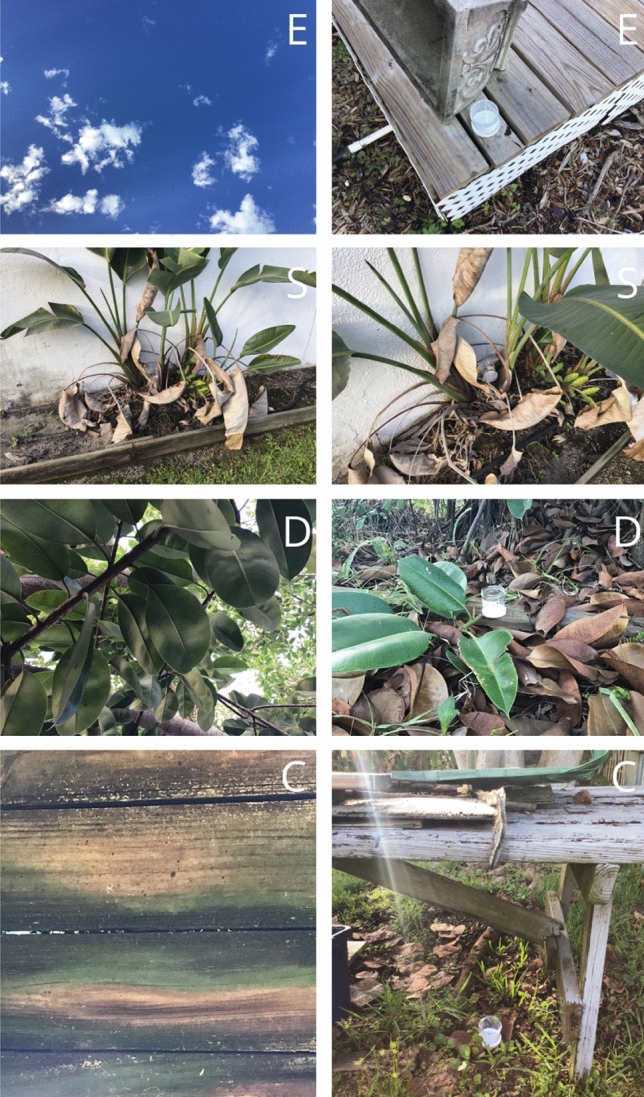
Sampling location coverage type in the treatment site. From top to bottom: (E) Exposed to sky, (S) sparse vegetation cover, (D) dense vegetation cover, and (C) covered (obstructed from sky)

To assess the WALS performance, we used bioassay jars dispersed across the target areas to test the efficacy of the droplets generated by the equipment during each spray. We selected 20 residential parcels within our treatment site and 10 parcels within the control site for placement of bioassay jars (Fig. 3). Within each parcel of the treatment site, dry and open 177 ml polystyrene bioassay jars were placed in the front and back yard of each home, on average 9 m and 25 m from the spray path, respectively. The control site had one jar in each home approximately 16 m from the street. Each bioassay jar was placed randomly under one of four spatial scenarios: Exposed to the sky, in sparse vegetation, in dense vegetation or in a covered location (completely obstructed from the sky, Fig. 4). The placement of the bioassay jars was to simulate the presence of *Ae. aegypti* larval habitats and the potential for the droplet cloud to fall in those habitats [30, 31]. A total of 50 polystyrene 177 oz bioassay jars per replicate were placed in the field the afternoon of application at fixed locations. The jars were left overnight to ensure full droplet dispersal throughout the treatment site and to follow the operational procedures of Manatee County for any given spray run.

The morning after WDG application, between 7:00 and 8:00 AM, the bioassay jars were picked up, covered and brought back to the district. As previously described, the jars were filled with 100 ml of reverse-osmosis water and mixed to suspend *Bti* residue. Approximately 17–20 L3 lab-reared *Ae. aegypti* larvae were then added to each jar (treatment and control) to record larvae mortality per jar at different times post -treatment (1 h, 3 h, 24 h and 48 h after exposure). Larvae that could not resurface or did not react to probing were considered dead.

### Ovitrap surveillance

Throughout the trial period, egg and larval surveillance data was collected in both the treatment and control sites. Seed germination paper (Nasco, Fort Atkinson, WI) was clipped to 473 ml black oviposition jars (Ball Mason Jars spray painted with matte black) and the jars were filled with approximately 250 ml of reverse-osmosis water that was infused with 5 g of the 3:2 liver and brewer’s yeast powder mix. We deployed 25 oviposition jars (15 treatment sites, 10 control sites, Fig. 3 [32, 33]. The ovitraps were placed in a shaded area of the yard and remained in the designated location throughout the trial period. The oviposition papers were collected and changed weekly to coincide with adult surveillance. Broken or missing jars were replaced as required. Egg papers from each ovitrap were placed in labeled plastic containers to maintain humidity and limit egg desiccation while in transit back to the laboratory. There, the number of eggs was counted under a dissection microscope and recorded. Positive egg papers were individually submerged in mosquito breeders (BioQuip^©^ Products, Inc., Rancho Dominguez, CA) with 100 ml reverse-osmosis water and were fed 1 ml of the 3:2 liver yeast slurry every other day until pupation (approximately 7 days). As soon as the adults emerged, the mosquitoes were frozen, identified and counted.

### Adult surveillance

To measure adult population dynamics, we used Biogents (BG) Sentinel 2 Traps [1] baited with BG Lures (Biogents AG, Regensburg, Germany) and landing rate counts (LRC). To compare sites to assess the effect of VectoBac WDG application, variance was minimized by determining fixed collection sites for the treatment and control study sites instead of sampling randomly across both sites each week [33]. The collection sites were chosen in the treatment and control sites by looking at past adult surveillance data and based on the knowledge of *Ae. aegypti* flight range being approximately 100–500 m [34]. A total of 20 traps were deployed weekly in the treatment (10 traps) and control sites (10 traps [Fig. 3]) for the duration of the trial period and LRCs were conducted at each location twice weekly and averaged to get the mean LRC for that study week. To perform an LRC, a designated person stood in a location for a total of three minutes and counted each *Ae. aegypti* that landed [35, 36]. The three-minute protocol was used to account for a noticeable delayed landing/biting behavior of our local *Ae. aegypti.* The choice of fixed surveillance was initiated by asking permission from residents. We gave each resident a brochure with information regarding the study and in cases where the residents were absent, we left a door hanger with our contact information. The BG traps were deployed continuously for a 24 h period in the chosen yards (Fig. 3). Surveillance with the traps was typically in a shaded and /or vegetated area due to *Ae. aegypti* resting preferences of cool, low to the ground dwellings and proximity to shelter [37]

### Data analysis

Bioassay container larval mortality was calculated by converting the total number of dead larvae in the bioassay jars at 48 h to percent larval mortality for all application days (*n* = 8) which were combined and analyzed using a one-way analysis of variance (ANOVA).

The effect of *Bti* application in the treatment site was investigated by comparing the number of eggs collected from ovitraps, the number of adults captured by the BG-Sentinel traps and biting pressure at the treatment site versus the untreated control site. Over-dispersion is common in count data sets such as these and many studies focused on mosquito populations recommend Poisson regression for analyzing such over dispersed data [38, 39]. The data were analyzed using a Poisson regression in ‘R’ software (Table 1 [40]). The average was calculated per week for each site (untreated control, WALS) and plotted for all the variables using Ggplot2 in ‘R’ [41].

## Results

### Open-field droplet analyses and bioassays

**Fig. 5:**
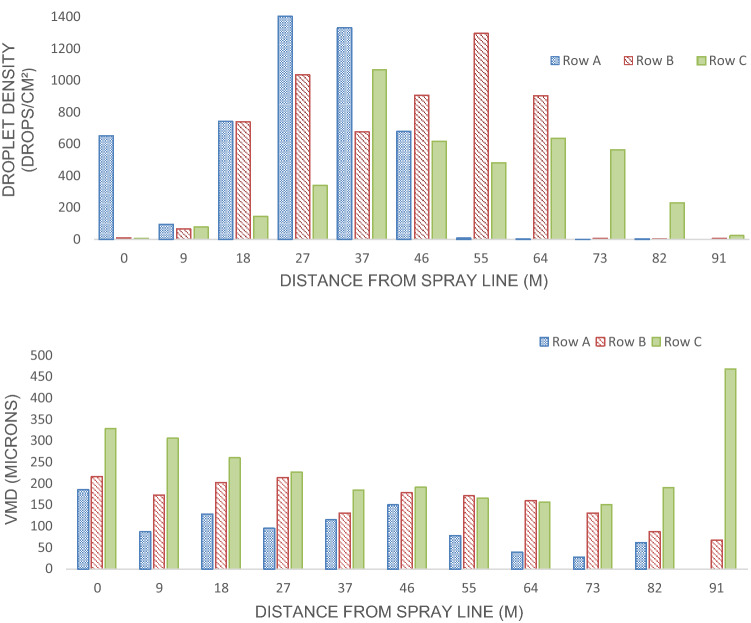
Volume density and droplet sizes of VectoBac WDG applied using a truck-mounted A1 Super Duty Mister sprayer at 560 g/ha in an open field

The size and droplet density were monitored in an open-field setting with Kromekote cards. Mean droplet sizes (VMD) for rows A, B and C were 185 µm, 157 µm and 238 µm, respectively. Generally larger droplets settled out closer to the spray line. Additionally, droplet density was measured and as the droplets traveled through the open-field test grid the droplet density increased up to 64 m. There was a direct relationship between droplet density and distance from the spray path, as the distance increased 64–91 m, the droplet density decreased. The number of droplets ranged from 2–1,401 droplets/cm^2^ in Row A, 3–1,293 droplets/cm^2^ in Row B and 7–1,067 droplets/cm^2^ in Row C (Fig. 5).

**Fig. 6:**
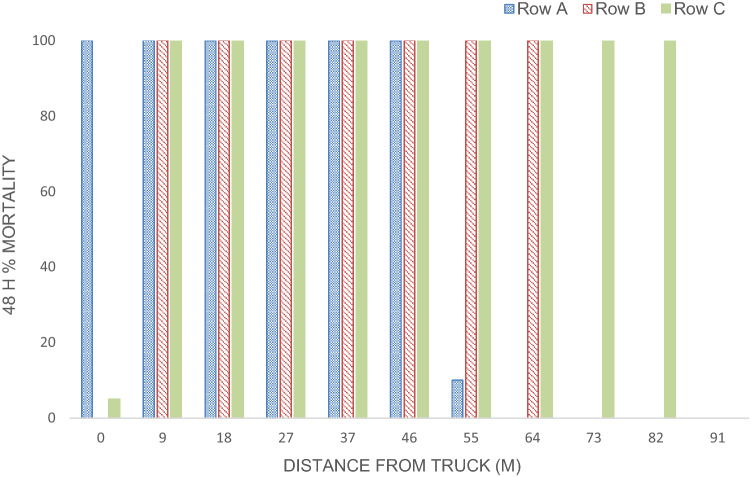
Droplet characterization: A1 Mister VectoBac WDG application open-field trial larval bioassay using L3 *Aedes aegypti* (USDA) June 3, 2020 Sunny Shores, Cortez, Florida

Bioassays that were conducted in conjunction with the droplet characterization indicated that the A1 mister WALS application of *Bti* resulted in larvicide deposition throughout the open-field test grid, with an average mortality of 69.86 ± 6.1% out to 91 m for all three rows (A, B and C). When data are further examined, the average mortality was greatest (100%) for all three rows at distances 9–46 m with a mean VMD of (218 µm). At 64 m from the spray line, rows B and C reached 100% mortality (mean VMD = 158). For row C, droplets (mean VMD = 215 µm) at distances 64–82 m still resulted in high mortality (90.5 ± 2.73%). However, the droplets (mean VMD = 84 µm) of rows A and B at distances of 73–91 m resulted in 0% mortality, row C also reached 0% mortality with droplets (VMD = 467 µm) at 91 m (Fig. 6). There was no mortality in the control bioassay jars that were placed upwind of the spray path. Weather conditions during the droplet characterization trial averaged 23.89 °C, 88% RH and 10.94 km/h wind speed.

### Operational trials

The study included 16 weeks of consistent surveillance that corresponded to the epidemiological weeks 21 to 38 in the county’s calendar. WALS applications with Vectobac WDG took place on weeks 23—26, 28, 30, 32 and 34. Application day was initially included as a variable in the ANOVA model but there was no overall significance compared to the coverage type and location of bioassay jar, therefore it was dropped from the final model. The interaction between treatment, coverage type and location in yard was not significant (F_3_ = 0.28, P = 0.840) which indicates that regardless of where the bioassay jars were placed, the mortality was similar. There was a significant difference (F_1_ = 355.4, P =  < 0.0001) in mean larval mortality averaging about 77% ± 1.9 in the treatment site compared to the untreated control site (< 0.21%, Table 2).

**Fig. 7:**
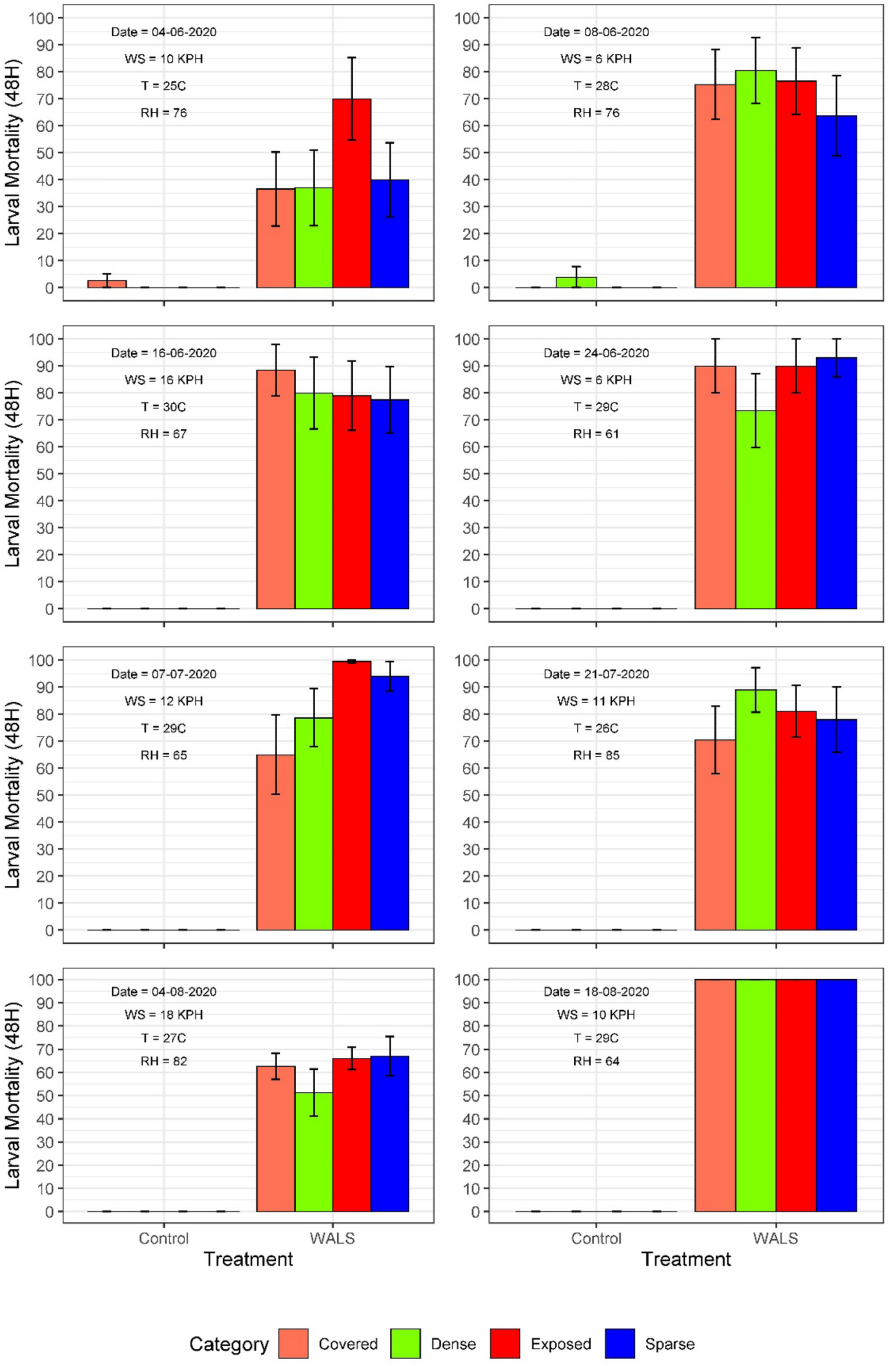
Percent larval Mortality (±SE) one-way analysis of variance (ANOVA): L2/L3 *Aedes aegypti *larval bioassays after weekly/biweekly A1 Mister VectoBac WDG applications during May–August 2020, Manatee County, Florida

The efficacy of the WALS application in Cortez, FL; when measured through the bioassay jars, demonstrated a similar average larval mortality for each coverage type (exposed to sky, in sparse vegetation, in dense vegetation or in a covered location). The mean mortality from the eight applications was calculated and in the bioassay jars that were covered or obstructed from the sky in the front and back yard was 70.59 ± 3.41% and 76.96 ± 2.59%, respectively. In the bioassay jars that were placed under dense vegetation, the mean larval mortality for the jars in the front and back yard was 82.02 ± 2.52% and 66.20 ± 3.7%, respectively. Similarly, the bioassay jars that were placed in areas completely exposed to the sky in the front and back yard saw an average mortality of 89.13 ± 2.4% and 78.77 ± 2.7%, respectively (Fig. 7). Under sparse vegetation, the average larval mortality in the bioassay jars in the front and back yards was 85.71 ± 2.6% and 65.81 ± 3.9%, respectively (Fig. 7).

### Effect of WALS application on population dynamics

**Fig. 8:**
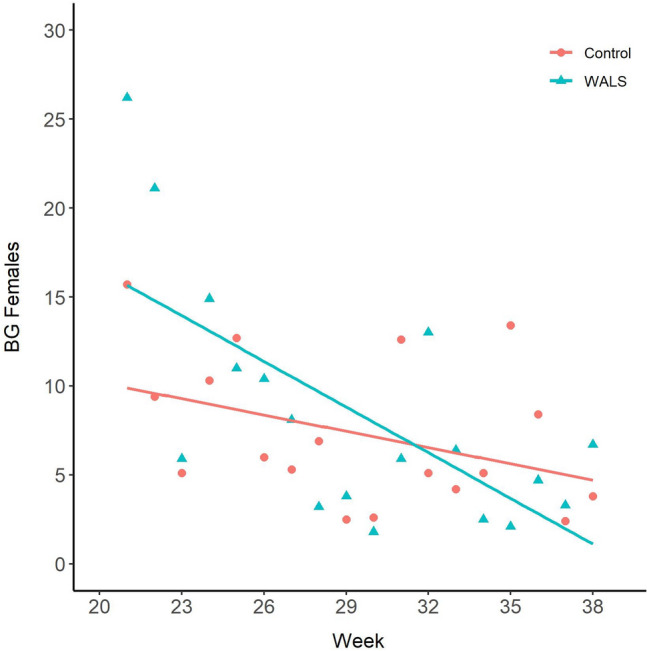
Average females caught in BGS traps for each week for untreated control and WALS sites

**Fig. 9:**
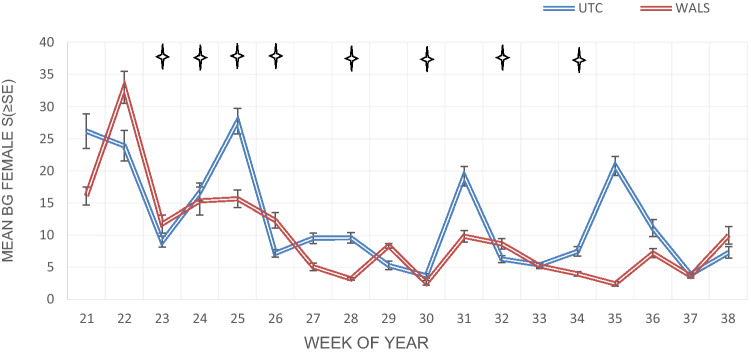
Mean Total female and male *Ae. aegypti *collected from the BG-Sentinel 2 trap in the untreated control (Blue) and the treatment (Red). Star indicates Vectobac WDG application

**Fig. 10:**
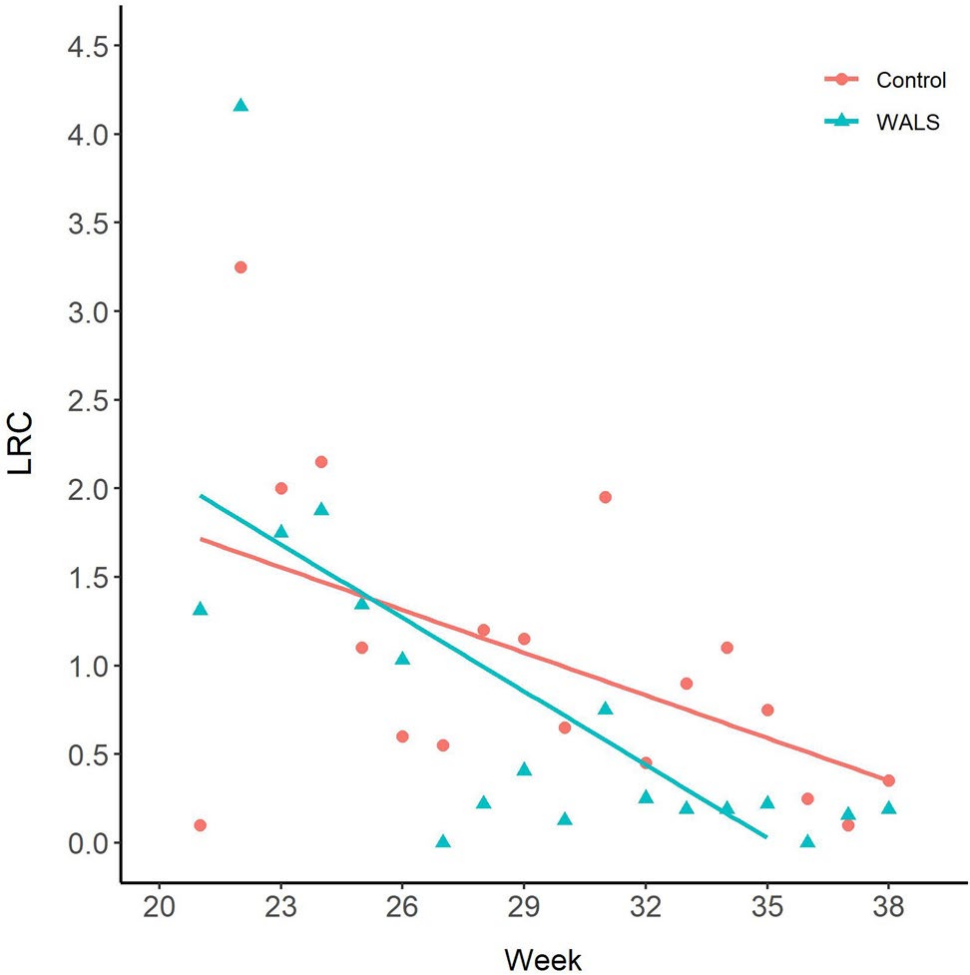
Average number of females collected (Landing Rate Count, LRC) for each week in untreated control and WALS sites

**Fig. 11:**
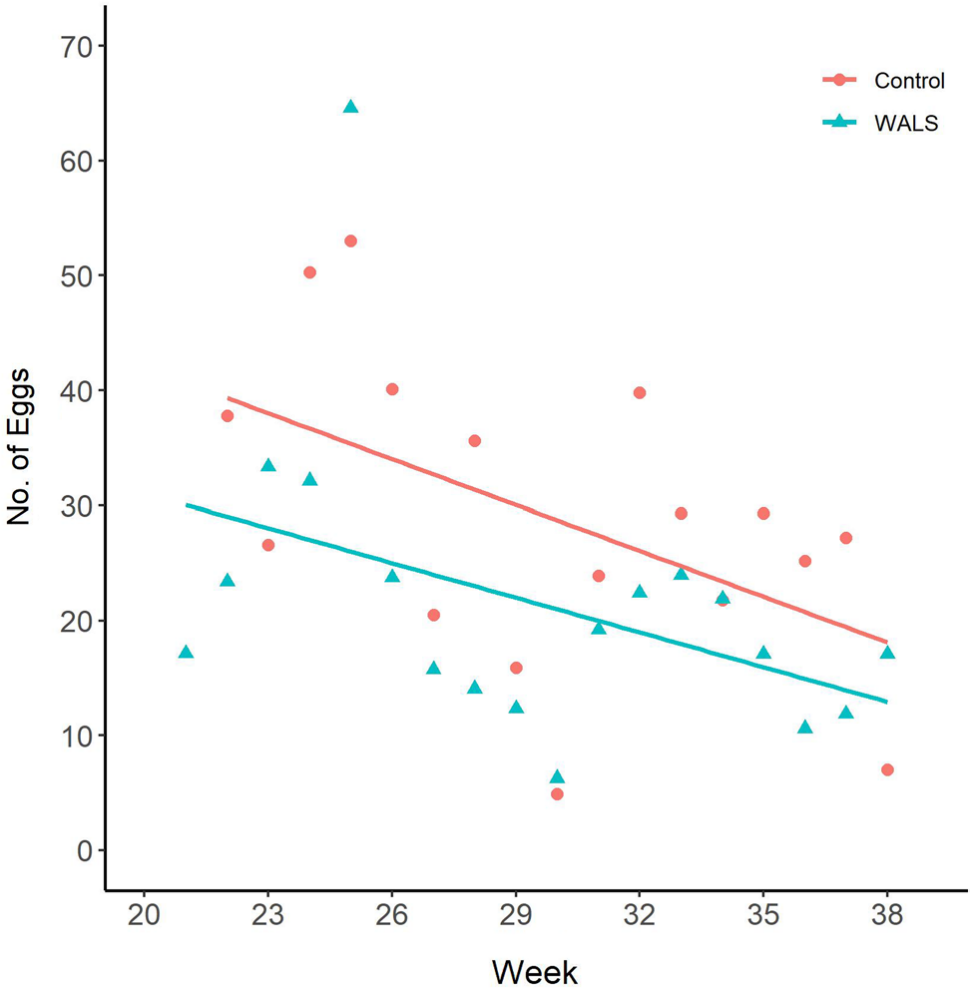
Average number of eggs collected from the ovitraps for each week in untreated control and WALS sites

Overall, there was a general decline in adult numbers in both the WALS and untreated control sites (Figs. 8 and 9). This is the typical trend every year as the abundance of *Ae. aegypti* decreases in the later months in Manatee County. Regardless, *Ae. aegypti* female abundance caught in the BG-Sentinel trap was significantly reduced in the WALS site compared to the untreated control site (P = 0.0002). Similarly, in the WALS site a significant reduction in biting pressure (Fig. 10) was observed compared to the untreated control site (P = 0.0058). Unlike female abundance and LRC, the eggs collected from ovitraps in the WALS site did not show a decrease in trend compared to the untreated control; however, there was a significant difference (P =  < 0.001) in the number of eggs collected from the WALS site compared to the untreated control site (Fig. 11).

## Discussion

We assessed the WALS strategy utilizing the A1 Super Duty Mister to spray *Bti* as a means of reducing *Ae. aegypti* populations in Cortez, Florida, which was ultimately an attempt to manage domestic mosquito populations throughout Manatee County. Our results indicated that areas with limited access such as private properties, have been found to benefit from wide-area *Bti* applications as they target both natural and artificial containers such as flowering pots, shriveled foliage, drains and spots below houses [15] and have the potential to thoroughly cover the backyard and habitats where the larvae can be found [20, 27].The benefits of using a *Bti* product like VectoBac WDG in this manner is twofold as it is a bacterium found naturally in soil that has proven to be a predominant biological control measure [42]. The efficacious bacterium produces a crystalline protein inclusion which is toxic to mosquito larvae upon ingestion [25, 43]. *Bacillus thuringiensis israelensis* can be used to target the larvae of both mosquitoes and black flies and is non-toxic to other insects, invertebrates, and vertebrates [15, 42, 43, 44]. In addition to its environmentally friendly nature, the risk for resistance to *Bti* is considerably low and therefore can be utilized in a resistance management program [44]. Local populations of *Ae. aegypti* have been investigated in separate studies by Manatee County to determine the susceptibility status of this species to insecticides of common use. The most recent field trials showed that certain pyrethroids resulted in only a 10% overall mortality in local *Ae.* aegypti populations which is an indicator that other control strategies are needed (data not shown).

Ultra-low volume (ULV) sprayers are a preferred method of adulticide applications due to the high-pressure systems generating small droplet sizes that can be carried slowly at great distances through the air to make contact with its flying target [24]. When conducting ULV applications with adulticides, it is optimal to produce droplets for ground applications that are less than 30 µm [45]. Unfortunately, these smaller droplets are more susceptible to wind conditions as they will tend to drift long distances, possibly missing the intended target and often impacting the efficacy of a spray mission [26]. The objective of larviciding for container mosquitoes is to make droplets large enough to fall into larval container habitats but small enough to drift short distances. To generate the appropriate flow rates and droplet spectrum, truck mounted, or aerial low volume (LV) mist sprayers can be utilized to produce larger droplets (LV) into the air which will facilitate droplets large enough to come down rather than drift long distances compared to ULV generators and backpack sprayers [26].

Like all insecticide applications, calibrating equipment to produce the correct range of droplets is imperative for a successful treatment. In the open-field analysis, droplets that caused over 70–100% morality in any of the rows had a mean VMD between 158 and 218 µm. Other successful truck-mounted mist applications have noted reduced *Aedes* populations when the droplet range was between 111 and 232 μm in areas that would otherwise be challenging to control with conventional mosquito control methods [26, 30, 46]. Conversely, a mean VMD of ≤ 84 μm or ≥ 467 μm resulted in almost no mortality. Although small droplets are ideal for ULV applications, here the larval habitats require ≥ 80 μm for maximum efficacy [26, 27].

In open-field trials the expected decrease in mortality with increasing distance from the spray line was observed, with greater deposition and observed larval mortality up to 64 m from the spray line. However, 100% mortality and large droplet size (VMD = 238 μm) were observed in row C (final row in the spray line), this is most likely due to the wind direction (ESE-E) at the time of application and the fact that the truck was driven 121 m after row C, allowing droplets to be propelled more uniformly throughout the row. This dispersal of the droplets was also facilitated by the wind created by the mister and the wind speed of 10.94 km/h were sufficient to push the droplets through the test grid.

The purpose of the open-field trials was to test the efficacy of the A1 mister and its ability to provide larval control under ideal conditions without the presence of obstructions such as tree canopy, houses and fences. However, residential areas such as Cortez, FL house a variety of landscapes that all provide prolific breeding sites for disease carrying *Aedes* mosquitoes [15, 22, 43] and applications targeting the anthropophilic *Aedes* are typically conducted in these suburban or urban settings [44]. Administering *Bti* to the larvae, commonly found in hidden receptacles or water collecting litter on private property, requires diligent habitat identification and frequent access to yards.

The WALS application in one treatment site in Cortez, FL resulted in a significant difference in mean larval mortality 77% (P =  < 0.0001) in comparison with the untreated control (0.21%). The consistent results after each of the eight operational trials suggest that this strategy of *Bti* application has the potential to be used effectively in residential areas and that the WDG spray can reach a variety of egg-laying habit regardless of obstruction as well as coverage type. However, it was observed that while some sites reached full mortality in hours, some bioassay jars under the same coverage and location in the yard struggled to obtain a similar level of mortality. This could be due to the layout of buildings and landscapes which potentially affected the spray cloud and obstructed the product from reaching the larvae. Two different bioassay jars treated under identical conditions, under sparse vegetation for example, can reach different levels of mortality [30, 31]. Wind direction and wind speed could have also impacted mortality in the bioassay jars. The truck drove in the same direction each application night and the bioassay jars were placed in fixed locations; however, the wind direction and speed varied which could account for a lower observed mortality on some nights. On the last application spray event (week 34), 100% mortality was observed in all the bioassay jars. The greater efficacy on that evening is most likely due to the rainfall that occurred (7.1 cm) after the WALS application. Potentially, *Bti* was washed into the jars from the leaf canopy, roofs, rain gutters and tabletops leading to increased control. This indicates that a rain event before an application could be used to maximize coverage in these larval habitats. There was no significant difference in mortality based on the location of the jars in relation to whether the jars were placed unobstructed from the sky, sparsely obstructed, densely obstructed, or completely covered (P = 0.840). Similar results were reported by Harris et al. [30] where they tested the WALS strategy in Puerto Rico under various types of vegetative cover.

To assess the effect of the WALS applications on *Ae. aegypti* adult populations in Cortez, BGS traps were deployed and monitored weekly. We found a significant reduction in female adults in the treatment site compared to the untreated control site. These results were supported by a reduction in egg production in ovitraps as well as a decrease in biting pressure during the application period. The number of eggs produced in the WALS treatment site was significantly reduced; however, the overall trend in the treatment and control sites similarly decreased over time. This could be due to variations in adult populations and egg-laying habitat between sites. The treatment site had a difficult sampling location where egg production was noticeably higher serving as a hot spot. This location had three jars that were under heavy canopy where debris would fall in. The breakdown of plant material and other microorganisms provides a nutrient rich environment that can cue gravid *Ae. aegypti* to oviposit [47]. Additionally, a high number of eggs collected in the ovitraps in the treatment site can be attributed to *Bti* serving as an attractant to some *Aedes* species, such as gravid *Ae. albopictus* [48, 49]. While egg production showed a similar trend over time in both sites, we recorded a decrease in landing rate counts in the treatment site demonstrating that the WALS applications significantly impacted adult female *Ae. aegypti* populations.

Other operational studies have demonstrated the WALS strategy as efficacious to control *Aedes* populations. Williams et al. [26] conducted urban and suburban applications of Vectobac WDG to reduce *Aedes albopictus* populations employing similar truck-mounted equipment and reached high larval mortality and efficacious control of the species in municipalities of New Jersey. In addition, operational research in Florida by Pruszynski et al. [31] showed that a series of WALS applications with aerial equipment significantly decreased adult female populations of *Ae. aegypti* in Key West by > 50%.

Meteorological factors such as rainfall, temperature and relative humidity affect mosquito oviposition activity which can be measured in positive ovitraps and egg density [50]. Throughout the present study, the average rainfall during the months of April- August was unexpectedly low in 2020 at just 33% of the average rainfall for this time of year in Manatee County (mean = 0.13 cm, 198.6 mm total rainfall) when compared to 2019 (mean = 0.43 cm, 598.9 mm total rainfall). As a result, the local populations of *Ae. aegypti* may have exploited the ovitraps deployed in the treatment and untreated control sites and therefore the reduction of adult female *Ae. aegypti* observed in the treatment site was not reflected in egg and larval collections. Additionally, we cannot discount adjacent untreated areas and potential migration of adult mosquitoes which can disrupt proper reduction in the number of eggs, larvae and adults found in the traps [43]. It is also important to note that this trial was carried out starting in the last two weeks of May with our first application occurring on the evening of June 4, 2021. The delayed start and short baseline monitoring period (two weeks) were due to the COVID-19 pandemic that created personnel restrictions and county-wide closures of businesses across the state. According to Manatee County’s historical data, the season for *Ae. aegypti* begins in May and starts to slow down in August each year. However, with the drier season, mosquito numbers were comparatively low in 2020 making it difficult to determine if there was any difference in egg production.

On three occasions during the trial (Weeks 23 and 33), residents of the untreated control site called in a service request regarding large numbers of mosquitoes in their yards. Mosquito Control technicians responded by applying DeltaGard® at the mid-label rate of 1 g of deltamethrin/hectare using a Maruyama MM300 backpack mister (Maruyama, Fort Worth, TX). Application of DeltaGard can cause adult mosquito mortality within 10 to 15 min of application, therefore impacting the number of adults laying eggs and flying into traps up to 15 days post application [5]. Although larval control measures were successful in significantly reducing the female adult population of *Ae. aegypti* mosquitoes in the treatment site, adulticiding activities potentially reduced the number of adults in the untreated control site making it difficult to interpret variables such as egg production and number of larvae present.

Despite these limitations a significant difference in adult population between the treated and untreated control site was observed demonstrating the WALS approach as an effective tool for the control of *Ae. aegypti* and other container-inhabiting mosquito species. However, it should be noted that successful mosquito control operations expect a higher percentage of control in local adult populations. The WALS approach should be utilized in conjunction with an adulticide program to furtherincrease mosquito population decline.

Studies exploring the use of backpack sprayers and misters have found that area-wide applications of *Bti* to target *Aedes* mosquitoes can be a cost-effective way of reaching egg-laying sites [43, 16], and additionally in highly urbanized settings it was significantly less expensive and strenuous than source reduction. There are many formulations and application methods of *Bti* and while each have their benefit, reaching cryptic *Ae. aegypti* breeding habitat is a top priority. VectoBac® water-dispersible granule (WDG) is an optimal formulation for this container mosquito, where the spores can reach the mosquito habitat through an aqueous spray mix [30]. However, it is costly and future studies need to be done to test the efficacy at a lower rate to make it a viable option for districts to utilize in an IVM program.

## Conclusions

The WALS method of wide-area larviciding to reach difficult to control domestic mosquitoes was tested and the results are promising. Some adult populations have been found to recover 15 × faster than neighboring areas that had no *Bti* applications [46]. Therefore, investigating the residual properties of *Bti* products and appropriate timing is imperative to effectively integrate applications in densely populated areas with high levels of *Aedes* populations. Integration of new approaches and control methods such as the one presented here will be a viable option in addition to traditional adulticiding approaches used in established IVM programs [15, 46]. Cost-effectiveness must be further evaluated before regular applications can be introduced into our vector control program.

**Table 1:** Mean and standard deviation for all variables tested. The ‘Poisson’ column provides the p values for significance test comparing untreated control to WALS site for all variables tested

Adult Measurement Variable	Treatment	Mean	Standard Error	Poisson (*P* Value)
BG Females	UTC	7.3	9.1	0.0002
	WALS	8.3	16.6	
Eggs	UTC	28.7	44.5	<0.0001
	WALS	21.4	33.5	
LRC	UTC	1	1.7	0.0058
	WALS	0.7	2

**Table 2:** ANOVA table for larval bioassay data. Treatment: Control and WALS, Category: exposed, dense, sparse or covered

Variable	DF	F value	*P* Value	Mean Mortality
Treatment	1	355.4	<0.0001	Control 0.212 WALS 76.7
Category	3	1.2	0.326	
Treatment*Category	3	0.28	0.840	

## Supplementary Information

Below is the link to the electronic supplementary material.

Supplementary file1 (DOCX 21 kb)

## Data Availability

Data supporting the conclusions of this article are included within the article. The datasets required to reproduce analyses and results presented herein are available from the corresponding author upon reasonable request.
