# Supplementary material for: Field evaluation of WALS truck-mounted A1 super duty mist sprayer® with VectoBac® WDG against Aedes aegypti (Diptera:Culicidae) populations in Manatee County, Florida
Source: SN Appl Sci. 2022 Jan 6;4(2):50. doi: 10.1007/s42452-021-04893-x (PMC8733432; doi:10.1007/s42452-021-04893-x)
Supplement: Supplementary file 1 — Supplementary file1 (DOCX 21 kb) [file 42452_2021_4893_MOESM1_ESM.docx]

**Additional File 1**

**BacDrop Droplet Analysis**

1. Droplet Analysis using BacDrop™
   1. Scan the Kromekote cards and make into image files onto a file in the computer. Scanner is a Fujitsu ScanSnap S 1300i (Fujitsu Computer Products of America, Sunnyvale, CA).
   2. Open the BacDrop program, enter the parameters for graphs (enter color code number for color of dye, enter size of Kromekote card used and how far apart the cart stations were established
      1. Red dye=color code 220, 70 x 90 mm cards set 9 m apart.
   3. Hit “Submit” details. The program will read each card and create three documents.
      1. Document 1=Excel Raw Data
      2. Document 2=VMD and Droplet Density Graph
      3. Document 3=VMD and NMD graph.
   4. Files are dropped into the folder where the card images are stored.

**Additional File 2**

**Manatee County Mosquito Control District Insectary Procedures**

**INTRODUCTION**

**The Manatee County Mosquito Control District rears field collected eggs, larvae and adults for resistance testing and bioassays. Adults are blood fed to maintain a colony up to F3 or F4. In addition, the District rears mosquitoes from eggs or larvae of susceptible colonies that are maintained at the USDA.**

**2. Insectary**

2.1.1 Environmental Conditions

2.1.2 Equipment and Supplies

- 1. **Field Collection of Mosquitoes**

2.2.1 Ovi-Jars: *Aedes aegypti, Aedes albopictus,* during summer-fall

2.2.2 Gravid Traps & Larval Dipping at Waste Management Plant: *Culex quinquefasciatus*

- - 1. Inspector collections throughout county: *Aedes taeniorhynchus*

**2.3Larval Rearing & Adult Care**

- - 1. Larval Food for different species
    2. Pupation
    3. Adult Feeding

2.3.4 Blood-feeding

**2.1.1**

**Environmental Conditions**

Temperature:

Set at 28^o^C and maintained by a Trane^®^ Cooling Air Conditioner and Heat Pump. The 20”x20”x1” filter should be changed at least every 6 months.

Humidity:

Controlled by an AIRCARE Space Saver Evaporative Humidifier Model 831000. The 1043 Superwick^®^ filters should be replaced every 6 months.

Desired Conditions:

Larval rearing: Water temperature of 78-80^°^ F

Adult maintenance: Air temp 75-80^°^ F

Relative Humidity: 78%

When rearing for special projects, the conditions may change according to the requirements of the project.

**2.1.2**

**Equipment and Supplies**

Adult Cages:

Collapsible Cage 12” x 12” x 12” aluminum frame w/20x20 mesh screening: Bioquip^®^ #1450B without nylon feeding hammock

8” Stockinette material cut to 16” lengths for the 12x12x12 cage: Derma Sciences 79408 Cotton Stockinette, 8”x25 yd, Amazon # 79408

Screen spline 0.160”

No-See-Um 20x20 Fiberglass screening 72”x25’ : Phifer Home Depot 4829/K8M

Larval Rearing Trays:

Clear Sterilite Trays, 23- 1/2” x 16- 7/8” x 5-7/8” Wal-Mart

Food, Larval:

Liver Powder (Bovine): MP Biomedicals^™^ Liver Powder, Fisher Scientific #90039601

Brewer’s Yeast: MP Biomedicals^™^, Fisher Scientific #90031205

Other: Rabbit Food, Hog Chow, Fish Food

Food, Adult:

Table sugar, e.g., Dominoes or store brand

Filter paper: GE Healthcare Whatman^™^ Qualitative filter paper: Grade No. 1 circles,

125mm diameter, Fisher Scientific #09805F

Water:

Purified water Reverse Osmosis unit: Kent Marine RO50TFC Champion Lighting Co., 1 micron Sediment Cartridge #1MS, 1 micron Carbon Cartridge # 1MC, and 50 GPD RO Membrane #MEM50

Salt: Canning & pickling salt

**2.2**

**Field Collection of Mosquitoes**

Eggs are collected with ovi-jars & gravid traps, larvae are collected via dipping as required for the study.

- - 1. Ovi-Jars collect mosquitoes seeking an oviposition site. Using a 3:2 liver yeast slurry as attractant and food, they have historically been used to catch *Ae. aegypti* and *Ae. albopictus.*
    2. Gravid traps collect mosquitoes seeking an oviposition site. Using hay water infusion (“stinky water”) as the attractant, they have historically been used to catch Cx*. quinquefasciatus* at Orbans, Lena Road, and at the District shop.
    3. Inspector collections/Larval dipping throughout the county for *Ae. taeniorhynchus* and *Culex spp.* throughout the county.

**2.3**

**Larval Rearing & Adult Care**

2.3.1 Larval Food for Different Species:

Liver Powder and Brewer’s Yeast are stored at room temperature, dry, or as indicated by the manufacturer.

Mix or prepare larval food per specifications for the specific species.

*Aedes*-3:2 liver/yeast slurry (500ml of RO water, 15g liver/10g yeast), Feed days 1,3,5 (30 ml-50 ml)

*Culex-*same as above

*Anopheles-* 1:1 liver/yeast slurry, dust powder of hog chow and fish food in equal amounts, 1:1:1 liver/yeast/hog chow slurry. All foods are fed on different days depending on development, Feed days 1 & 3- (1:1), days 4 & 6- (dust), days 5 & 7 -(1:1:1).

(25-50 ml)

Refrigerate slurries.

Rabbit food or hog chow can be stored at room temperature or refrigerated.

2.3.2 Pupation:

Manual Pupae Separation, remove pupae using a disposable pipette from rearing pan and place them in emergence cup. Place rearing cup in mesh cage with a vial filled with sugar solution and filter paper.

2.3.3 Adult Feeding:

Adults are fed a 10% sugar water solution, using 80 grams sugar/800 mls RO water. Label and date the container. Keep the sugar water solution refrigerated. If mold develops, wash the container well with soapy water containing bleach, using a brush to dislodge the mold. Rinse thoroughly after cleaning.

To feed the mosquitoes, roll a filter paper and placing it in a 40 ml amber vial containing the 10% sugar water. The sugar water will wick up the filter paper, and the mosquitoes will land on the filter paper to feed.

2.3.4 Blood-feeding :

1 bottle of 250 ml Defibrinated Bovine Blood: Hemostat Laboratories #DBB250

Bemis Parafilm M^™^ Laboratory Wrapping Film: Fisher Scientific #13-374-12

Equate Jumbo Cotton Balls (400 count): Wal-Mart

Oven to warm blood ball for 45 minutes: VWR^®^ Signature Forced Air Safety Oven.
